# A rare presentation of nonbacterial thrombotic endocarditis

**DOI:** 10.1016/j.jvscit.2026.102337

**Published:** 2026-05-26

**Authors:** Clement Jose, Paul Andre, Jeremiah Langsfeld, Megham Brett, Ross Clark, Mark Langsfeld

**Affiliations:** a Department of Surgery, University of New Mexico Hospital, Albuquerque, NM; b Department of Cardiology, University of New Mexico Hospital, Albuquerque, NM; c School of Medicine, University of New Mexico, Albuquerque, NM; d Department of Infectious Diseases, University of New Mexico Hospital, Albuquerque, NM

**Keywords:** Endocarditis, Ischemia, Embolization

## Abstract

Nonbacterial thrombotic endocarditis is a rare disease in which a hypercoagulable condition—usually malignancy or an autoimmune state—precipitates the formation of sterile vegetations on cardiac valves. Mortality is high due to cerebral embolization, cardiac failure, and progression of the underlying disease process. Lower-limb ischemia is uncommon, and reported upper-limb and hepatic ischemia are rare. Both transthoracic and transesophageal echocardiography are the diagnostic modalities of choice for confirming valvular vegetations. We report a patient who presented with multiple unusual embolization sites; with multidisciplinary assistance, she was diagnosed and treated for nonbacterial thrombotic endocarditis.

Nonbacterial thrombotic endocarditis (NBTE) is a thrombotic disorder that occurs in hypercoagulable patients and usually involves the left-sided heart valves. In 1936, Gross and Friedberg^1^ published a thorough classification system and description for this unusual disease process that previously was mainly diagnosed at autopsy. Subsequently, Macdonald and Robbins^2^ noted the various organs involved with embolic infarctions and the grave consequences of this disease. Vascular surgeons normally do not manage these patients primarily, but may be called on to treat patients who present with limb or organ ischemia. The majority of these patients present with cerebral emboli and/or cardiac symptoms. However, patients with NBTE may also present with visceral ischemia (renal, splenic) and, less frequently, with lower limb ischemia.^3^, ^4^, ^5^, ^6^, ^7^ Even more rarely, they may exhibit hepatic ischemia or upper limb ischemia.^6^ We describe a case where vascular surgery was consulted on a patient who presented with simultaneous upper limb, lower limb, and hepatic ischemia. The goal of this article, therefore, was to further educate vascular surgeons on the potential presentation, diagnosis, and management of patients with this unusual disease process.

## Case report

The patient was a 63-year-old woman who had no previous known medical history and recently travelled to our state via a cross-country airplane ride. She presented to our Level 1 trauma center following 2 days of intermittent pain and weakness in her right lower and upper extremities. The emergency department initially considered diagnoses of stroke, venous thromboembolism, and lower limb ischemia, so she was sent for emergent computed tomography (CT) scan of the head, chest, abdomen, and lower-extremity runoff. There was no acute intracranial process, but a large right breast mass was noted on CT (Fig 1), along with thrombus in her common hepatic artery, as well as a splenic infarct (Fig 2). At this point, there was concern that the breast mass could be a malignancy and discussion to complete an oncologic workup, including a biopsy, was proposed. However, given the presence of acute lower limb ischemia, the workup of this mass was deferred until the life-threatening pathology was addressed first. The CT also demonstrated a pulmonary right upper lobe suprahilar irregular nodule with soft tissue attenuation measuring 2.0 cm, along with an adjacent peripheral nodule measuring 2.4 cm. This was among several other small pulmonary nodules. The initial CT scan of the chest did not mention findings of a mitral valve lesion on the final read. Although this was not a gated study and increased contrast burden on the left-sided chambers, there was not a clear visualization of a vegetation.

**Fig 1:**
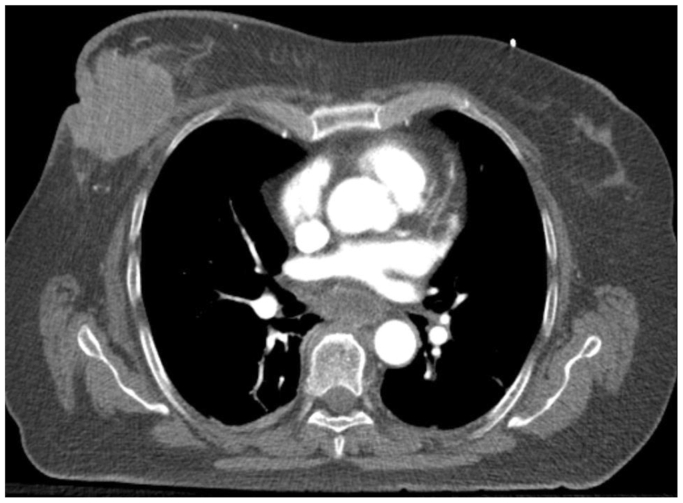
A representative slide from a computed tomography (CT) scan showing the large right-sided breast mass.

**Fig 2:**
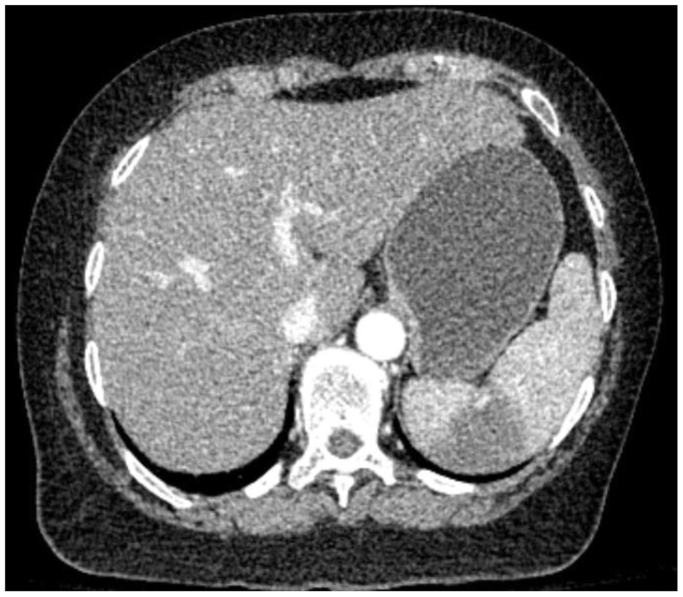
A representative slide from a computed tomography (CT) scan showing the splenic infarct.

On return to the emergency department, she had increasing right leg and right arm pain, decreased right foot motion, and was noted to have absent Doppler signals in her right leg. Review of the CT scan showed right common femoral artery occlusion (Fig 3). Vascular surgery was consulted, and she was diagnosed with acute Rutherford 2B ischemia to both her right arm and right leg. On physical examination, her abdomen was soft and nontender, and ischemic right upper and lower extremity findings were confirmed. The patient was started on heparin and emergently taken to the operating room. Embolectomies successfully re-established blood flow via right groin and right antecubital incisions. Fasciotomies were then performed in the right calf. Because her serum lactate was normal, no abdominal exploration was performed at that time.

**Fig 3:**
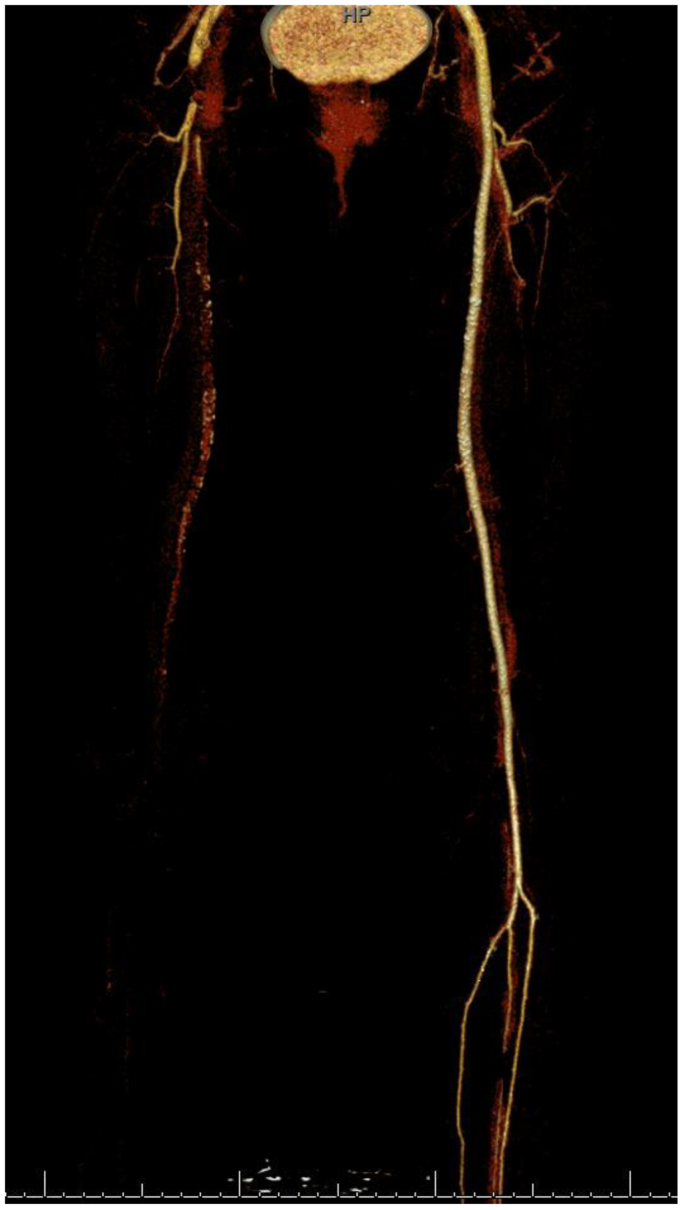
Extremity runoff showing right common femoral artery occlusion and no distal flow.

During her intensive care unit stay the next day, heparin anticoagulation was continued. Her electrocardiogram showed no atrial fibrillation. A transthoracic echocardiogram (TTE) was performed. It was considered technically difficult, but a nodular, thickened surface on the anterior leaflet of the mitral valve was noted. The patient then started developing abdominal pain, and vascular surgery took her back to the operating room for abdominal exploration and hepatic revascularization. An embolectomy of the common hepatic and gastroduodenal arteries was performed, along with a cholecystectomy. The embolectomies were performed using a single arteriotomy of the common hepatic artery just proximal to the gastroduodenal artery takeoff. Using a combination of 2F and 3F Fogarty catheters, embolectomies were performed of the common hepatic artery, gastroduodenal artery, proper hepatic artery, right and left hepatic arteries separately, and the celiac artery. Given the identification of hepatic and gallbladder ischemia on workup, these were the only embolectomies considered during this index mesenteric revascularization procedure. Postembolectomy Doppler ultrasound examination of the liver parenchyma show arterial signals throughout and inspection of the liver demonstrated a well-perfused appearance.

The largest size of the clot retrieved through the embolectomies was 1.5 cm × 1.5 cm; however, this was sent as a conglomerate of the embolectomies from the multiple mesenteric sites. The clot was acute appearing with a homogenous appearance throughout. The borders of the clot were unable to be accurately assessed due to the use of a Fogarty catheter for embolectomies.

The abdomen was then packed open and the patient taken back to the intensive care unit for supportive care. Over the next several days, she was taken back to the operating room for abdominal closure as well as fasciotomy closures. The hematology, oncology, infectious disease, and cardiology services were consulted and extensive laboratory studies were ordered. Over her hospital course, all blood cultures and other laboratory studies such as antiphospholipid antibodies and lupus anticoagulant were negative for infection, prothrombotic, or rheumatologic disorders. Blood cultures included fungal cultures and workup also included a respiratory panel, which were all negative. Postoperatively, the patient had return of sensory function in the form of intact sensation to touch, temperature, and pain and slowly improving motor function over the course of 4 weeks. Her extremities were able to be salvaged, and amputation during her hospitalization was avoided. The patient required physical and occupational therapy due to the significant physiological insult from multiple surgeries and her underlying pathology. Per American Society of Hematology recommendations for patients with thromboembolic events and presumed breast cancer, she was discharged on oral apixaban to an inpatient rehabilitation facility.

The patient was readmitted approximately 1 week later with significant fatigue and a workup for sepsis ensued. Her temperature was 37.8°C, with a white blood cell count of 22,000, hemoglobin of 6.6, and lactate of 4.9. CT of her chest was performed showing bibasilar ground-glass and consolidated opacities concerning for aspiration or infective bronchiolitis. The previously noted right breast mass and right-sided pulmonary lesions remained unchanged, and there were no pulmonary emboli. All previous and repeated blood cultures remained negative. Group B *Streptococcus* pneumonia was diagnosed and treated with eventual clinical improvement.

She also underwent biopsies and was diagnosed with invasive ductal carcinoma of the breast with metastases to the lymph nodes and lung. Her final cancer diagnosis was an estrogen receptor/progesterone receptor-positive, HER2-negative invasive ductal stage 4 breast cancer. Extensive hematologic workup for a hypercoagulable state and infective endocarditis was performed and all studies were negative. Pathology on all previous embolectomy specimens showed organized thrombus without evidence of inflammation or bacterial clumps. A repeat TTE during her second admission showed a 2.1 cm × 1.6 cm mass on the mitral valve (Fig 4). This was confirmed on a transesophageal echocardiogram (TEE), along with significant mitral regurgitation (Fig 5). The final diagnosis per hematology was NBTE secondary to a hypercoagulable state due to invasive and metastatic breast cancer. She was started on letrozole for the cancer and was eventually discharged to an inpatient facility.

**Fig 4:**
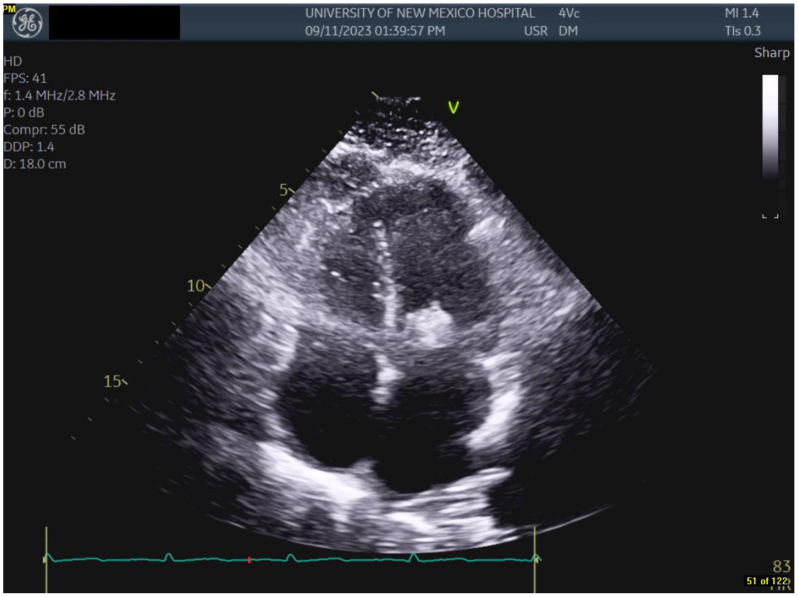
Transthoracic echocardiogram (TTE) apical four-chamber view with a large mass on the mitral valve.

**Fig 5:**
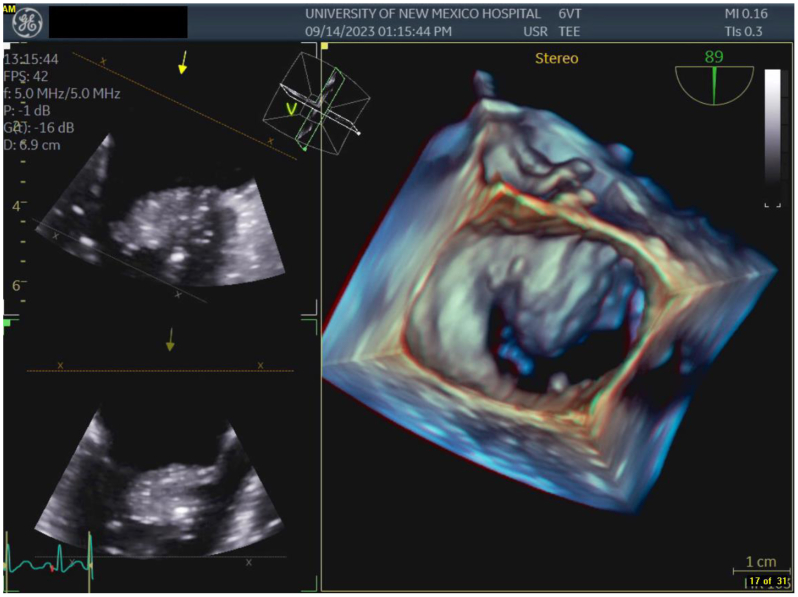
Transesophageal echocardiogram (TEE) three-dimensional assessment of the mitral valve in diastole showing large mass on the A2 leaflet of the mitral valve.

The patient was readmitted 1 month later with signs of congestive heart failure. Both palliative care and hospice were consulted, and she declined aggressive treatment of her breast cancer. She was discharged after a 4-week hospital stay. Before discharge, the patient agreed to publish case details and images for educational purposes. However, after discharge, she never followed up with multiple scheduled clinic visits and contact with her and family has been unsuccessful. Therefore, her long-term functional status could not be assessed.

## Discussion

NBTE is a disorder rarely managed by vascular surgeons. Patients present with a hypercoagulable state, usually due to end-stage carcinoma, or patients may have lupus or antiphospholipid antibody syndrome. Other described disorders associated with NBTE are burns, rheumatoid arthritis, sepsis, and COVID-19 infection.^8^^,^^9^ Patients typically have friable, sterile vegetations composed of fibrin and platelets on previously nondamaged left-sided heart valves, most commonly the mitral valve, and may present with cardiac symptoms as well. The fibrin and platelet clots are postulated to result from endothelial dysfunction from the underlying hypercoagulable state and resulting circulating immune complexes.^7^, ^8^, ^9^, ^10^

Most patients are asymptomatic, but when they develop symptoms, they usually present with stroke symptoms from cerebral emboli or with cardiac symptoms such as chest pain, dyspnea, or heart failure. Less commonly, other peripheral emboli can occur, such as renal, splenic, or lower extremity emboli. Multiple emboli signify presentation in late stages of disease with a hypercoagulable state.^11^ Once the ischemic beds are managed, anticoagulation is paramount during the workup for the source of the emboli and underlying condition. Currently, there are no formal recommendations on anticoagulation with direct oral anticoagulants for NTBE, so heparin, warfarin, and aspirin continue as the mainstay agents, depending on the underlying disease process. Warfarin is the treatment of choice for patients with antiphospholipid antibody syndrome.^9^ Although the routine use of anticoagulation in NBTE is not recommended, there are specific indications for its use. Based on the 2020 American Heart Association guidelines, there are no clear recommendations for the management of NBTE in patients with endocarditis. However, they emphasize that management is focused on the underlying etiology. This should include anticoagulation when managing the underlying malignancy or autoimmune disorder that is driving the prothrombotic state.

The diagnostic criteria used for the patient presented here are the guidelines from the European Society of Cardiology diagnostic algorithm for NTBE.^9^ These criteria identified patient clinical factors such as malignancy or autoimmune conditions and clinical presentation, including evidence of stroke or systemic thromboembolism. Patients meeting these factors will then receive a TTE. If vegetations are not identified, then the patient will require a TEE.

Our patient presented with systemic emboli and a mass consistent with cancer. TTE identified vegetations. If vegetations are identified, then infectious endocarditis should be ruled out. This evaluation was completed with our infectious workup. Her hypercoagulable state was identified and the diagnosis of NBTE was confirmed. The patient met all criteria for a workup for this diagnosis throughout the hospital stay. Patients with NTBE must be differentiated from those patients with infective endocarditis or blood culture-negative infective endocarditis. Patients with infective endocarditis usually present with fever or other signs of infection, and have risk factors such as valvular heart disease, prosthetic heart valves, recent vascular catheter placement, intravenous drug use, or intracardiac devices. These patients can also present with peripheral emboli, as noted in a recently published 8-year study of 525 patients with infective endocarditis, where 14% of patients had peripheral emboli, with splenic and renal vessels as the most common sites of embolization.^12^ Blood cultures may be negative in ≤30% of patients with infective endocarditis, so a search for exposure to nonculturable organisms must also be performed. Persistent fever with negative cultures or exposure to a recent antibiotic course just before blood cultures may indicate culture-negative endocarditis.^13^

Both TTE and TEE are essential to confirm the diagnosis of NBTE and to evaluate the extent of the cardiac disease. TEE is more sensitive than TTE in detecting valvular lesions, but often TTE is more readily available. In a 20-year study from the Cleveland Clinic, the diagnostic utility of echocardiography was validated after reviewing >600,000 TTE and 89,000 TEE studies that diagnosed 42 cases of NTBE. TTE diagnosed NTBE in 45% of cases, and TEE diagnosed 97% of cases in which it was used (33/34). The most common valves involved were the mitral (62%) and aortic (24%). Additionally, they identified that the most common pattern seen was a “vegetation-like echodensity, which appeared as a localized mobile echogenic mass attached to a valve, usually with thickened leaflets, particularly at the tips.” Sizes of the vegetations ranged from <1 cm to >2 cm. Serial cardiac echocardiographic studies may be necessary if already embolized cardiac vegetations are not initially seen, as was possible in our patient. Serial studies can show changes in the extent of vegetations as well as further assess cardiac function. The detection of valvular vegetations along with the presence of malignancy or autoimmune disease are strong indicators of NBTE.^14^

Because NBTE is uncommon, most of the recent literature involves reviews of case reports. In a systematic review of case reports and case studies between 1975 and 2018, Venepally et al^11^ reported on 163 patients with newly diagnosed NBTE. The mean patient age was 46 years, and 69% were female. The in-hospital mortality rate was 30%, and the most common presentation was stroke (54%). Acute limb ischemia was the reason for presentation in only 9 of the 163 patients (5.5%), although there was no mention of the site of the embolization. Malignancy was seen in 47% of patients, with antiphospholipid syndrome in 36% and systemic lupus erythematosus in 22%. Including the initial presentation and hospital course, 69.9% of patients had stroke episodes, 19.0% had splenic and renal infarcts, 18.5% developed heart failure, 15.0% had myocardial infarctions, and 12.0% had acute limb ischemia. The authors emphasized the importance of early suspicion and diagnosis of NBTE with anticoagulation to prevent morbidity and mortality. They emphasized that early treatment is critical to prevent further valve damage and embolization.^11^

In a recently published systematic review and meta-analysis, Alhuarrant et al^6^ reviewed 450 patients with NBTE in 416 case reports. The most common chief complaints on presentation were stroke-like symptoms (36%), chest pain and/or dyspnea (29%), and heart failure symptoms (10%). Documented peripheral emboli were very common, with 57% of patients having strokes, and 19% and 18%, respectively, having renal and splenic infarcts. Only 4% of patients had limb/digit ischemia, with no mention of any patient with upper extremity emboli. The most common etiologies for the hypercoagulable states were cancer (46%) and autoimmune disorders (42%). The all-cause in-hospital mortality rate was 36%, with higher mortality rates in patients >50 years or whose admission was complicated by the occurrence of emboli and cancer as the etiology of their hypercoagulable state.^6^

NBTE is an uncommon disorder, and previously has been mainly diagnosed at autopsy. In a review of 819 autopsy cases of both infective endocarditis and NTBE, none of the 404 NTBE cases were identified before death.^15^ However, with the increased availability and performance of both TTE and TEE, the antemortem diagnosis of this disease process has been increasing. Infective endocarditis can also present with embolic complications, but the diagnosis of NBTE is confirmed by echocardiographic findings of thrombotic vegetations on heart valves and negative blood cultures. Because the vegetations of NBTE are absent of inflammation at the valve site and are more friable than infective endocarditis vegetations, they are more susceptible to embolization. Our patient presented with multiple sites of embolization, but to our knowledge upper-extremity emboli have not been previously reported with NBTE. It is possible that prior case series reporting limb ischemia may not differentiate lower-limb from upper-limb involvement. Furthermore, with less muscle mass and more extensive collaterals in the upper limbs, it is possible that embolization to the upper limbs was not recognized clinically.

Unfortunately, once the clinical presentation occurs, NBTE is often end stage, resulting in high morbidity and mortality. Clinicians must have a high level of suspicion for NBTE, and aggressively use echocardiography in making the diagnosis as well as anticoagulation during the workup. A multidisciplinary approach is critical to differentiate NBTE from infective endocarditis and develop a treatment plan for the underlying causative disease process. Also important is monitoring of the patient's cardiac function with possible surgery needed for progressively deteriorating cardiac valves or the removal of persistently embolizing vegetations.^16^

## Conclusions

NBTE is a rare but often fatal disease that was previously only recognized at autopsy, but is now being diagnosed antemortem due to increasing awareness and utilization of echocardiography. Patients are often asymptomatic until they present with cerebral embolic phenomenon and/or cardiac symptoms. Multiple unusual sites of embolization, as occurred in our patient, often portend end-stage underlying conditions, such as malignancy. This was the case in our patient; she did not present with the typical signs of cerebral ischemia and/or cardiac symptoms, but with unusual locations of simultaneous, symptomatic emboli. Vascular surgeons must be aware of this disease process in their workup after treating peripheral ischemia and maintain anticoagulation therapy during the postsurgical evaluation process. In this case, we received valuable input and management from the medical oncology, hematology, infectious diseases, cardiology, cardiac surgery, pulmonary, and palliative care services. Thus, a multidisciplinary approach is critical in the management of these unique and complicated patients with NTBE; cardiac problems can progress and underlying malignancy and autoimmune disorders must be diagnosed and treated.

## Funding

None.

## Disclosures

None.
